# Associations between adolescents’ use of sexually explicit material and risky sexual behavior: A longitudinal assessment

**DOI:** 10.1371/journal.pone.0218962

**Published:** 2019-06-26

**Authors:** Goran Koletić, Taylor Kohut, Aleksandar Štulhofer

**Affiliations:** 1 Department of Sociology, Faculty of Humanities and Social Sciences, Zagreb, Croatia; 2 Department of Psychology, University of Western Ontario, London, Canada; University of Westminster, UNITED KINGDOM

## Abstract

The unprecedented accessibility and affordability of online sexually explicit material (SEM) has facilitated widespread use among adolescents and growing concerns over adverse reproductive health outcomes. Although SEM-induced risky sexual behavior is among key concerns, there is a paucity of longitudinal research addressing this issue. This study aimed to assess the longitudinal association between frequency of SEM use and risky sexual behavior among adolescents in two independent 5-wave panel samples of Croatian adolescents (*n* = 368; *n* = 247). The indicators of risky sexual behavior were: (1) not using a condom at most recent sexual intercourse, and (2) reporting two or more sexual partners. Multilevel logistic regression analysis with a lagged component was used to explore the associations of interest. Controlling for sociodemographic characteristics, pubertal timing and sensation seeking, frequency of SEM use was not associated with the two indicators of sexual risk taking. This study’s findings are relevant for health and educational experts, but also for concerned parents.

## Introduction

In the last two decades, the proliferation of Internet use and advances in supporting technologies have made sexually explicit material (SEM) widely available and increasingly accessible [1]. Unsurprisingly, this has resulted in widespread SEM use among adolescents [2,3]. Such dynamics have triggered considerable concerns among parents, policy makers and the scientific community about potentially harmful outcomes associated with SEM use. Among these concerns, suggested links between SEM and adverse adolescent health outcomes—particularly in the form of sexual and reproductive health risks—occupy a central position [4].

Risky sexual practices among adolescents include early sexual debut, which has been found to be systematically associated to reproductive health risks, such as unprotected sexual intercourse (sometimes as the consequence being intoxicated while having sexual intercourse) and multiple sexual partners [5]. Among risky sexual behaviors, unprotected sexual intercourse is often the center of attention because it increases one’s risk of acquiring a sexually transmitted disease or experiencing an unwanted pregnancy [6]. Engaging in unprotected sexual intercourse is ranked second among the leading health risks for both male and female adolescents globally [7]. In the past 15 years, a substantial proportion of adolescents in Central and Eastern Europe have acknowledged engaging in risky sexual behaviors, which has been associated with an increase in sexually transmitted diseases other than HIV, as well as with unabated rates of teenage pregnancies and abortions [8,9]. These public health issues have prompted research into psychosocial and cultural influences on adolescent sexual risk taking [10,11]. However, the potential role of SEM use has received limited attention in this context.

Existing research concerning the relationship between SEM use and risky sexual behavior among adolescents is characterized by mixed findings. Two American cross-sectional studies, one conducted only among African-American girls aged 14–18 [12] and another among adolescents aged 12–22 [13] found a positive association between SEM use and multiple sexual partners. A Swiss cross-sectional study conducted among adolescents aged 16–20 years by Luder an her collegues [14], however, failed to corroborate this relationship. Furthermore, within these studies a significant association between SEM use and not using a condom at most recent sexual intercourse was found among female adolescents [12] in one case but only among male adolescents in the other [14]. Neither of these findings were corroborated by a more recent cross-sectional study using a sample of Australian participants aged 15–29 [15]. Two longitudinal studies have examined the relationship between SEM use and risky sexual behavior among adolescents. A two-wave study of Dutch adolescents aged 12–17 found no association between SEM use and casual sexual intercourse without using a condom [16]. But again, a more recent three-wave study among Canadian adolescents (aged 13–17 at baseline) found that regular SEM users were more likely to report more sexual partners, compared to low frequency SEM using adolescents [17].

Several differences among existing studies may be the reason for mixed findings regarding adolescents’ SEM use and sexual risk taking. Firstly, studies differ based on sampling strategies. Three studies used convenience-based samples [12,13,15] and three used nationally representative samples [14,16,17]. Secondly, mixed findings may reflect differences in study samples in regard to age range. Thirdly, there were notable differences in measures of SEM use. For example, Lim and her colleagues [15] measured frequency of SEM in the last 12 months, Peter & Valkenburg [16] in the last 6 months and Rasmussen & Bierman [17] measured frequency based on the number of pornographic videos seen in the last year. Finally, much of available research is cross-sectional in design, therefore precluding a possibility to assess direction of the target association. In this context, longitudinal designs may be particularly useful because they can examine associations over longer periods of time, include both time-variant and time-invariant covariates, control for previous sexual risk behavior and frequency of SEM use, and explore potential changes in risky sexual behavior as a function of SEM use.

### Theoretical framework

Research concerning the effects of SEM use among adolescents is characterized by substantial theoretical and conceptual diversity, which reflects multidisciplinary nature of the field. In this study, we used the recently developed Acquisition, Activation, and Application Model (_3_AM) of adolescents’ and adults’ sexual media socialization [18]. The model is based on the sexual scripting theory [19] that views sexual behavior as personally, inter-personally and socio-culturally scripted. According to the _3_AM [18], SEM-generated sexual scripts, which are commonly focused on lust, pleasure and unprotected sex with multiple sexual partners [20], may influence attitudes toward sex and serve as guidelines or cues for an actual sexual behavior. By being exposed to such scripts, adolescents may find sexual practices portrayed in contemporary SEM—which rarely show any risks—normative, common, and even rewarding. In addition, two developmental implications stem from the current theoretical framework. As suggested by the _3_AM, the scripting process would affect adolescents depending on their trajectories of SEM use [21] and the acquisition of SEM-related scripts during adolescence may shape patterns of risky sexual practices that would extend to emerging adulthood [22,23].

### Current study

Noting the lack of longitudinal assessments of the association between adolescent SEM use and risky sexual behaviors, the current study aimed to address the inconsistent findings in this area. To enhance robustness, we explored the target relationships in two independent panel samples of Croatian adolescents, with the smaller panel serving as a replication sample. This study’s findings are relevant for adolescent sexual and reproductive health specialists, sexuality and media literacy educators, but also policy makers and worried parents [2,4,24].

To control for potential confounders, we included several individual and family characteristics (sensation seeking, early pubertal onset and parents’ education) in our models. Sensation seeking has been repeatedly associated with adolescents’ SEM use [14,25], but also associated with increased likelihood of engaging in sexual risk taking [26]. By being more open to exploration, thrill, and novel sensations, adolescents who are high in sensation seeking may be more prone to risk taking. According to Zhang, Zhang, & Shang [27], lower assessment of the risk of adverse health outcomes seems to mediate this relationship. Adolescents who score high on sensation seeking are also more likely to use SEM, compared to their peers [28]. A recent meta-analysis found that pubertal timing was associated to risky sexual behaviors [29]. Early maturing adolescents were characterized by higher likelihood of engaging in risky sexual behavior. The mechanism underlying this association seems to be a combination of psychosocial and biological factors. Early maturing adolescents are more likely to engage in risky sexual behavior due to fewer parental restrictions, but also because their interest in behaviors of more mature individuals. [29]. In addition, early bloomers are more easily recognized as potential sexual partners by older adolescents or emerging adults, compared to their peers. We controlled for parents’ education, a common proxy for family socioeconomic status, because a low family socioeconomic status has been linked to multiple risk behaviors, including sexual risk taking, in adolescence [30].

#### Hypotheses

According to the _3_AM framework and previous research, we hypothesized that SEM use would be associated with adolescent sexual risk taking, such that: frequency of SEM use would be positively associated to reporting two or more sexual partners (H1) and the frequency of SEM use would be positively associated with reporting no condom use at most recent sexual intercourse (H2).

## Method

### Participants

Data for this study were collected as part of the PROBIOPS (Prospective Biopsychosocial Study of the Effects of Sexually Explicit Material on Young People’s Sexual Socialization and Health) research project which involved a longitudinal assessment of sexualized media use among adolescents. The study included two independent panel samples of Croatian high-school students: a 6-wave classroom-based panel in Rijeka (the third largest city in Croatia) and a smaller 5-wave online panel conducted in Zagreb (the capital city). Waves in both samples were spaced approximately 6 months apart.

The recruitment in Rijeka included 14 out of 22 high-schools. Seven smaller high-schools with less than 50 2^nd^ year students were omitted (due to financial reasons) and a school was left out because of a recent burglary and arson. The baseline survey in Rijeka was carried out during December 2015. Of the 2,074 2^nd^ year high-school students in the Rijeka, 1,307 participated at baseline sample (T0; aged 15–17; *M*_age_ = 15.83; *SD* = 0.52; 43.9% male students), *n* = 1,309 (T1), *n* = 1,252 (T2), *n* = 1202 (T3), *n* = 944 (T4), and *n* = 924 (T5). Data cleaning resulted in participant exclusion due to the following reasons: severely incomplete or invalid questionnaires (*n* = 139), reported decreasing or highly inconsistent number of lifetime sexual partners in the subsequent waves (*n* = 177) and participated only in one wave (*n* = 291). In addition, 302 students did not participate in the last two waves because they graduated a 3-year high-school program after T3. Overall, *n* = 1,071 (T0; aged 15–17; *M*_age_ = 15.82; *SD* = 0.49; 38.4% male students), *n* = 1,064 (T1), *n* = 1,031 (T2), *n* = 986 (T3), *n* = 775 (T4), and *n* = 749 (T5) participants were eligible for the analysis presented here.

To assess a potential bias introduced by attrition, multivariate logistic regression analysis was carried out with the dependent variable representing those who participated in all six data collection waves (0) and those who did not (1). All of the other indicators used in this study served as independent variables. Two significant differences were observed: male (*OR* = 1.48, *p* = .034) and older students (*OR* = 1.97, *p* = .045) were less likely to participate in all waves.

### Procedure

Prior to the onset of data collection, school headmasters distributed a letter to parents to inform them about the nature of the study. The data collection took place in the classrooms during school hours. We used a self-administered survey, with portable 50 x 50 cm screens separating students to enhance confidentiality. After providing information about the study, the study coordinator remained in the classroom until the survey was completed. A simple (unique) 5-letter identification code was used to link participants’ questionnaires across time. No incentives were used in the Rijeka panel. Informed consent was printed on the questionnaire frontpage and students were instructed to read the consent before proceeding further. Contact information for an online youth help service was provided at the end of each questionnaire.

### Replication sample

To enhance the robustness of our findings, the analyses were replicated in an independent online panel of adolescents from Zagreb, the Croatian capital city. The Zagreb panel was launched in April 2015 (for details, see [31]). The panel suffered from a high attrition rate from baseline (or T1) to T2—similar to the one reported in a recent online panel of Dutch adolescents [32]—but stabilized afterward. After the data cleaning, *n* = 1,057 (T0; aged 15–18; *M*_age_ = 16.14; *SD* = 0.45; 35.6% of male students), *n* = 636 (T1), *n* = 711 (T2), *n* = 683 (T3) and *n* = 686 participants (T4) were eligible for the analysis. Attrition bias assessment revealed that students who did not participate in all waves were more likely to be male (*OR* = 2.11, *p* < .001). Although the Zagreb panel was launched before the Rijeka panel, participants were roughly of the same age at baseline. Indicators used in the Zagreb panel were identical to those used in Rijeka. After providing informed consent by clicking on the appropriate button, participants were able to access an online questionnaire.

All study procedures for both panels were approved by the Committee on Ethics Issues in Science and Research of the Faculty of Humanities and Social Sciences, University of Zagreb. The Ethics Issues Confirmation was issued on 2^nd^ of June 2014. Informed consent procedure was reviewed and approved by the Committee. According to the national Guidelines for ethical research in minors [33], informed consent can be obtained from adolescents aged 14 years or older and parents had to be only informed about the research.

### Measures

#### Dependent variables

Two measures were used as indicators of risky sexual behavior: Condom use at most recent sexual intercourse (*Have you and/or your partner used a condom at most recent sexual intercourse*?) and having multiple sexual partners (*In total*, *with how many different people did you have a sexual intercourse since your first time*?). Using a branching question (*Have you had a sexual intercourse*?), only sexually active participants were asked to answer sexual risk taking questions. Both indicators of sexual risk taking were not assessed at the baseline (T0) of the Rijeka panel and therefore T1-T5 was the observed period. Following the literature [34], the number of sexual partners was dichotomized into 0 = one sexual partner and 1 = two or more sexual partners. Due to memory bias, condom use at the most recent intercourse is seen as a more precise indicator of sexual risk taking than asking about condom use over a longer period in the past [35].

#### Independent variable

SEM use was defined in the study as *any material which openly (i*.*e*., *not censored) depicts sexual activity*. *Material which shows naked bodies but not sexual intercourse or other sexual activity does not belong to pornography as here defined*. To avoid confusion, the term “pornography” was used in the questionnaires. Frequency of its use was measured at each wave with the item “*How often have you used pornography during the last 6 months*?*”* The response scale included the following options: 1 = *Never*, 2 = *Several times*, 3 = *Once a month*, 4 = *2–3 times a month*, 5 = *Once a week*, 6 = *Several times a week*, 7 = *Every day or almost every day* and 8 = *Several times a day*. The item was developed and used in several large-scale Croatian studies [26,36].

#### Control variables

Parents’ education was measured on a 4-point scale ranging from 1 = *unfinished primary school* to 4 = *college or university education*. Due to small percentages of parents with unfinished primary school and only primary school, the indicators were dichotomized into 0 = *college-educated* and 1 = *less than college-educated*. About one third of parents were college educated. Pubertal timing was assessed at the baseline by asking participants to estimate timing of their physical maturation, compared to their male/female peers. The response scale ranged from 1 = *much earlier* to 5 = *much later*. To pinpoint participants who reported earlier development, the indicator was dichotomized into 1 = *early maturing adolescents* (20.6% of male and 25.9% of female) and 0 = *others*. According to literature, self-reported pubertal timing is adequate for relative assessments [37]. Finally, sensation seeking was measured with the Brief Sensation Seeking Scale [38], which was developed and validated in adolescents. The 4-item measure was included in all waves in the Rijeka study (Cronbach’s *α* ranged from .75 to .83; stability coefficients were in the .62–.80 range).

### Analytical strategy

Students who participated in at least two of the five waves in total were eligible for the multivariate analysis. As the result of question branching, only adolescents who reported sexual intercourse were asked about the number of sexual partners and whether a condom was used at their most recent sexual intercourse. After sexually inexperienced adolescents were excluded, the model based on the Rijeka panel included 716 observations nested in 370 adolescents and model based on the Zagreb panel included 483 observations nested in 247 adolescents.

Multilevel random-intercept logistic regression analysis was used the explore the associations between SEM use and risky sexual behaviors. This analytical framework allows longitudinal assessments using unbalanced data (unequally spaced observations) with missing data [39]. Data preparation and modeling procedures were carried out following recommendations for multilevel modeling dichotomous outcomes [40–42]. The logit function served as the link function and robust covariance estimation method was used to test fixed effects. Models were analyzed using lagged approach; the outcome variable at time T+1 is predicted by SEM use at time T, while also controlling for the outcome at time T. Continuous independent variables were standardized to allow for comparisons of the effect sizes.

Model testing was carried out in three steps, separately by outcome and panel. First, the dichotomous outcome was regressed on a time variable to examine individual changes in the outcome. Time was significant for both outcomes among participants in both panels (the models are not presented in tables). In the second step, we regressed the outcome at T+1 on SEM use at time T, with autoregressive component included (Model 1). Finally, we assessed a full multivariate model, which included potential time-invariant (gender, parent’s education, pubertal timing and age) and time-varying confounders (sensation seeking) (Model 2). Due to gender-specific patterns of SEM use [21,28] and risky sexual behavior [8,34], a SEM use and gender interaction term was included in the final multivariate model.

To account for clustering effect in classes in Rijeka, we estimated intraclass correlations for both indicators of risky sexual behavior in the intercept-only models (results are not presented in tables). Classroom-based nestedness accounted for 6.6% of the variability in the odds of reporting two or more sexual partners and 2.6% of the variability in the odds of not using a condom. Due to small number of schools in Rijeka (*n* = 14), only classes were suitable for a higher-level analysis. In the Zagreb panel, clustering effect was assessed within schools. The respective percentages among female participants were 4.2% and 1.8%. Due to a reasonably small clustering effect in both panels, these higher-level effects were omitted from our multivariate regression models.

All analyses were carried out using IBM SPSS v24.

## Results

Proportions of adolescents who reported sexual intercourse, two or more sexual partners and not using a condom at most recent sexual intercourse per wave are presented in Table 1. At T1, 18.9% of adolescents from Rijeka and 16.8% of adolescents from the Zagreb panel reported having had sexual intercourse. Two years later, at T5, the proportion of sexually active adolescents from Rijeka and Zagreb increased to 47.4% and 42.1%, respectively. The proportion of adolescents who reported two or more sexual partners and engaging in unprotected sexual intercourse increased over time in both panels. On average, 41.8% of adolescents from Rijeka reported two or more sexual partners and 31.0% reported not using a condom at most recent sexual intercourse. Respective percentages for adolescents in the Zagreb panel were 42.7% and 30.4%. A more substantial increase in SEM use over time was observed in the Zagreb panel (*M*_T1_ = 2.97 to *M*_T5_ = 3.45), compared to the Rijeka panel (M_T1_ = 3.02 to M_T5_ = 3.09). Zero-order correlations between SEM use and the two indicators of sexual risk taking over time are presented in Tables 2 and 3. The associations, which were significant in 20 of 25 instances in the Rijeka panel and in 16/25 in the Zagreb panel, were positive and mostly of small size. In the both panels, correlations between SEM use unprotected sexual intercourse were mostly around zero.

**Table 1:** Key sexual behaviors by wave and panel sample.

	T1		T2		T3		T4		T5	
	*n*	%	*n*	%	*n*	%	*n*	%	*n*	%
Rijeka panel^a^										
Reported sexual intercourse	201	18.9	245	23.9	313	31.1	317	40.9	355	47.4
Reported 2 or more sexual partners	76	37.8	99	40.0	136	43.4	125	39.4	171	48.2
Reported condom non-use at most recent sexual intercourse	47	23.4	78	31.8	97	31.0	105	33.1	126	35.5
Zagreb panel^b^										
Reported sexual intercourse	178	16.8	183	28.8	189	26.6	255	37.3	289	42.1
Reported 2 or more sexual partners	72	40.4	73	39.9	73	38.6	118	46.3	139	48.1
Reported condom non-use at most recent sexual intercourse	46	25.8	43	23.5	54	28.6	90	35.3	112	38.8
	*M*	*SD*	*M*	*SD*	*M*	*SD*	*M*	*SD*	*M*	*SD*
Rijeka panel^a^										
SEM use (1–8)	3.02	2.35	2.82	2.22	2.97	2.27	3.01	2.29	3.09	2.32
Zagreb panel^b^										
SEM use (1–8)	2.97	2.41	3.19	2.39	3.23	2.35	3.30	2.34	3.45	2.31

^a^*n* = 1,064 (T1); 1,031 (T2); 986 (T3); 775 (T4); 749 (T5).

^b^*n* = 1,057 (T1); 636 (T2); 711 (T3); 683 (T4); 686 (T5).

To adjust wave notations, only the baseline in the Rijeka panel is named T0. Waves T1 to T5 denote the observed period in both panels.

**Table 2:** Cross-correlations between SEM use and the number of sexual partners by wave and panel sample.

	1	2	3	4	5	6	7	8	9	10
(1) SEM use T1		.78**	.78**	.72**	.68**	.15**	.21*	.16*	.28**	.34**
(2) SEM use T2	.86**		.83**	.79**	.73**	.14	.15	.13	.20**	.23**
(3) SEM use T3	.82**	.83**		.83**	.79**	.17	.12	.18*	.19**	.25**
(4) SEM use T4	.79**	.82**	.85**		.81**	.13	.09	.28**	.22**	.30**
(5) SEM use T5	.77**	.80**	.82**	.85**		.09	.07	.22*	.22**	.26**
(6) Sex. partners T1	.22**	.19*	.28**	.26*	.23*		.48**	.24*	.38**	.34**
(7) Sex. partners T2	.14*	.19**	.23**	.21*	.27**	.68**		.70**	.68**	.51**
(8) Sex. partners T3	.26**	.18**	.24**	.21**	.14	.60**	.83**		.65**	.55**
(9) Sex. partners T4	.14*	.02	.14*	.16**	.14	.50**	.65**	.77**		.80**
(10) Sex. partners T5	.17**	.12*	.11	.19**	.17**	.35*	.54**	.61**	.72**	

Zero-order correlation coefficients in the Rijeka panel are shown below the main diagonal, while coefficients in the Zagreb panel are presented above it;

* *p* < .05.

** *p* < .01.

**Table 3:** Cross-correlations between SEM use and unprotected sexual intercourse by wave and panel sample.

	1	2	3	4	5	6	7	8	9	10
(1) SEM use T1		.78**	.78**	.72**	.68**	-.01	.06	-.03	.13*	.01
(2) SEM use T2	.86**		.81**	.75**	.73**	.10	.05	.02	.13	.03
(3) SEM use T3	.82**	.83**		.83**	.79**	.05	.05	-.02	.13	-.06
(4) SEM use T4	.79**	.82**	.85**		.81**	.10	.01	-.04	.10	-.01
(5) SEM use T5	.77**	.80**	.82**	.85**		.07	.05	-.01	.15*	.02
(6) Condom non-use T1	.07	-.04	.20*	.08	.15		.39**	.38**	.26**	.31**
(7) Condom non-use T2	-.02	-.03	.03	.03	-.01	.43**		.52**	.34**	.42**
(8) Condom non-use T3	.11	.11	.06	.10	.12	.48**	.50**		.40**	.58**
(9) Condom non-use T4	-.12	-.05	-.01	-.04	-.09	.47**	.43*	.52**		.58**
(10) Condom non-use T5	-.11	-.03	-.01	-.04	-.01	.31**	.44**	.46**	.60**	

Zero-order correlation coefficients in the Rijeka panel are shown below the main diagonal, while coefficients in the Zagreb panel are presented above it;

* *p* < .05.

** *p* < .01.

### SEM use and multiple sexual partners (H1)

In the Rijeka panel, we observed no significant association between SEM use and multiple sexual partners (Table 4), either in the model without the controls (Model 1), or in the model with the controls included (Model 2). In contrast, adolescents in the Zagreb panel who used SEM more frequently had significantly higher odds of reporting two or more sexual partners (*OR* = 1.13, *p* = .036) than their peers who reported only one partner (Table 4). However, the association ceased to be significant after including the control variables (Model 2).

**Table 4:** Multivariate assessment of associations between the frequency of SEM use and reporting two or more sexual partners in the Rijeka and Zagreb panels.

	Rijeka Panel				Zagreb Panel			
	Model 1		Model 2		Model 1		Model 2	
	*OR*	95% CI	*OR*	95% CI	*OR*	95% CI	*OR*	95% CI
Multiple partners (at T-1)	28.58***	14.40–59.31	27.56***	13.81–57.79	32.26***	15.65–67.73	31.86***	15.68–68.38
SEM use (at T-1)	1.06	0.96–1.17	1.05	0.77–1.43	1.13*	1.01–1.28	1.31	0.94–1.73
Gender								
Female	–	–	1.24	0.16–8.84	–	–	2.45	0.41–14.70
SEM use & Gender								
Interaction	–	–	0.94	0.65–1.35	–	–	0.93	0.67–1.26
Mother’s education								
Less than college	–	–	0.69	0.40–1.17	–	–	0.96	0.50–1.84
Father’s education								
Less than college	–	–	2.07*	1.16–3.68	–	–	1.49	0.76–2.92
Pubertal timing								
Early maturing	–	–	1.50	0.87–1.13	–	–	1.22	0.69–2.18
Sensation seeking (at T-1)	–	–	1.06	0.99–1.13	–	–	–	–
Age (at T0)	–	–	1.32	0.75–2.33	–	–	1.45	0.77–2.71
Subjects (observations)	368 (712)		293 (578)		247 (483)		246 (481)	

SEM = sexually explicit material; CI = confidence interval around odds ratio (*OR*);

* *p* < .05,

** *p* < .01,

*** *p* < .001.

### SEM and condom use (H2)

In the Rijeka panel, we observed no significant association between adolescents’ frequency of SEM use and condom use at most recent sexual intercourse either in the predictor-only model, or the model with the controls included (Table 5). Gender did not moderate this association. Findings in the Zagreb panel fully replicated the results from the Rijeka panel.

**Table 5:** Multivariate assessment of the association between the frequency of SEM use and unprotected sexual intercourse in the Rijeka and Zagreb panels.

	Rijeka Panel				Zagreb Panel			
	Model 1		Model 2		Model 1		Model 2	
	*OR*	95% CI	*OR*	95% CI	*OR*	95% CI	*OR*	95% CI
Condon non-use (at T-1)	22.08***	14.21–34.32	17.59***	10.78–28.69	23.36***	13.81–39.53	20.54***	12.15–34.72
SEM use (at T-1)	0.95	0.87–1.04	0.99	0.78–1.28	0.97	0.88–1.08	1.02	0.84–1.24
Gender								
Female	–	–	1.47	0.30–7.10	–	–	1.41	0.40–5.00
SEM use & Gender								
Interaction	–	–	1.06	0.80–1.41	–	–	1.01	0.79–1.29
Mother’s education								
Less than college	–	–	1.16	0.76–1.77	–	–	1.80*	1.04–3.13
Father’s education								
Less than college	–	–	1.18	0.77–1.80	–	–	0.76	0.44–1.29
Pubertal timing								
Early maturing	–	–	1.14	0.74–1.75	–	–	1.38	0.85–2.25
Sensation seeking (at T-1)	–	–	1.01	0.95–1.07	–	–	–	–
Age (at T0)	–	–	0.85	0.57–1.26	–	–	0.94	0.55–1.63
Subjects (observations)	370 (716)		297 (579)		247 (483)		246 (481)	

SEM = sexually explicit material; CI = confidence interval for odds ratio (*OR*).

* *p* < .05,

** *p* < .01,

*** *p* < .001.

## Discussion

This study aimed to examine the association between adolescents’ SEM use and sexual risk taking. Prompted by the social and public health relevance of the issue, and the paucity of longitudinal studies focusing on this link [1,3,43], we used two independent longitudinal panel samples to examine the target relationship. With a number of potential confounders controlled for, a nonsignificant association between SEM use and multiple sexual partners was observed in the Zagreb and Rijeka panels (H1). We also observed a nonsignificant association between SEM use and unprotected sexual intercourse in both panels (H2).

The nonsignificant association between SEM use and odds of reporting two or more sexual partners is conceptually consistent with two cross-sectional studies [14,15], but direct comparisons are unwarranted due to methodological differences. However, our findings are in contrast with a recent Canadian prospective study [17] that found regular SEM users were more likely to report multiple sexual partners than low SEM users. The study’s age range and time span were substantially different from ours (participants in the Canadian study were aged 13–17 years at baseline and 18–23 years at the final wave), which prevents meaningful comparisons. Suggesting age-specific effects or, perhaps, that the influence of SEM use on multiple sexual partnerships requires a longer period of time to develop, a recent study by Braithwaite and his colleagues [23] conducted among emerging adults (18–25 years) also found a positive association between SEM use and the number of “hook up” sexual partners.

The non-significant association between the frequency of SEM use and unprotected sexual intercourse is conceptually consistent with the findings from a two-wave Dutch study [16] and two cross-sectional studies [13,15]. Interestingly, the same Dutch study found a positive association between SEM use and casual sexual intercourse without using a condom among participants aged 18 years or older. The finding is compatible with the hypothesis that the influence of SEM use on multiple sexual partnerships gradually develops over a number of years. This process may start with SEM use facilitating the endorsement of permissive sexual attitudes (including a preference for recreational and uncommitted sex) during middle to late adolescence, followed by an increased likelihood of engaging in risky sexual activity at a later developmental stage [44,45].

The “delayed effect” hypothesis seems plausible because the _3_AM model posits some audience factors (age, involvement and existing sexual scripts) and accessibility factors (recency, frequency and duration of media use) that may postpone sexual script acquisition and activation [18]. In addition, the notion of delayed effect is supported by the basic tenet of the cultivation theory [46]: exposure to pornography could subtly and over time cultivate viewers’ perceptions of sexuality. In this context, there is some evidence that timing of the first exposure to SEM is related to early sexual debut among male adolescents [47] and risky sexual behavior in emerging adults [36].

Although more cross-cultural studies will be needed to reach a conclusion, our findings do not support the possibility of a causal pathway between SEM use and sexual risk taking in the two middle to late adolescent panels. In addition, comparable prospective studies covering a period from adolescence to emerging adulthood may provide a more comprehensive insight into SEM-related sexual socialization and its possible behavioral outcomes.

Even if SEM use is unrelated to risky sexual behavior in most adolescents, it may influence adolescents’ sociosexual development in other ways. SEM often promote unrealistic sexual expectations, sexual objectification, and stereotypical gender roles [3,43]. The growing recognition of these issues prompted recent development and implementation of sexuality education programs in a number of countries [48–50] that include a pornography literacy module. In spite of the fact that there is evidence that such programs can assist young people in navigating through sexualized media landscape, potential opposition to their implementation—often based on the false premise that talking with adolescents about SEM means promoting its use [51]—may also need to be considered.

### Study strengths, limitations, and future directions

Several strengths and limitations of this study should be noted. Five measurement points enabled more precise analysis of conditional change in sexual risk taking, while the replication component, rare in behavioral research, added robustness. In contrast to previous studies, which were conducted in countries with a more liberal and permissive regulation of sexuality, ours was carried out in a country characterized by a strong Roman Catholic traditional [52] and restrictive approach to sexuality [53]. In such sociocultural context one might expect an attenuating or protective role of religiosity regarding SEM use [54]. However, religiosity seems to be unrelated to increasing SEM use [55] and only a weak evidence for a link between religiosity and sexual risk taking was found among Croatian youth [56]. Therefore, our findings may serve as culture-specific assessment of the relationship between adolescents’ SEM use and risky sexual behavior.

Several limitations should also be considered. Taking into the account the observed attrition bias, the findings in both panels may be more representative for female participants. Next, our replication sample lacked the time-variant measurement of sensation seeking. Further, due to different data collection methods, responses in our primary (classroom-based) panel sample were likely more vulnerable to social desirability than responses in the online panel. However, the effect would have been marginal as we observed no substantial differences in pattern of risky sexual behavior across samples. Our study did not include sexual orientation, because only a small number of participants reported same-sex attraction. Considering that non-heterosexual adolescents report more frequent SEM use than their heterosexual peers [15,28], especially male adolescents [14], and that positive associations between SEM use and risky sexual behaviors among adult gay men have been repeatedly reported [45], the importance of controlling for adolescents’ sexual orientation can hardly be overstated. Future research should also employ multiple measures of sexual risk taking, preferably composite indicators with high reliability. Last but not least, objective measures should be used instead of self-reports. For example, salivary testosterone assessment could be used to indicate pubertal development [57] or urine-based *Chlamydia trachomatis* testing as an indicator of sexual risk taking [12,58].

## Conclusions

Our findings expand the current knowledge about the association between adolescents’ SEM use and risky sexual behavior. Using longitudinal data spanning over a period of two years, we found no evidence of the role of SEM use in adolescents’ sexual risk taking in two independent samples. Considering the need to improve our assessment of potential confounders and moderators of possible links between adolescent SEM use and sexual risk taking, longitudinal analyses remain an imperative in this field.

## Supporting information

S1 Appendix Study measures. List of study measures as they were presented to participants.
(DOCX)

S2 Appendix Data set (Rijeka). (SAV)

S3 Appendix Data set (Zagreb). (SAV)
