# Supplementary material for: Associations between adolescents’ use of sexually explicit material and risky sexual behavior: A longitudinal assessment
Source: PLoS One. 2019 Jun 26;14(6):e0218962. doi: 10.1371/journal.pone.0218962 (PMC6594649; doi:10.1371/journal.pone.0218962)
Supplement: S1 Appendix — List of study measures as they were presented to participants. (DOCX) [file pone.0218962.s001.docx]

**Supporting information**

**S1 Appendix. Study Measures.**

List of study measures as they were presented to participants in both panel samples.

**Gender**

Your gender?

1 – Male

2 – Female

**Age**

Your birth month and year?

______(month); ______(year);

**Parents’ education**

**Your mother's highest level of education?**

1 – Unfinished primary school

2 – Primary school

3 – High school

4 – College

Your father's highest level of education?

1 – Unfinished primary school

2 – Primary school

3 – High school

4 – College

**Pubertal status (relative comparison; for boys and girls)**

In comparison to others, your physical development has:

1 – Began much earlier

2 – Began somewhat earlier

3 – Began about the same time

4 – Began somewhat later

5 – Began much later

**Sensation seeking**

| Estimate do the following statements relate to you: | IT DOES NOT RELATE TO ME AT ALL | IT DOES NOT RELATE TO ME | NOR IT RELATES TO ME NOR IT DOESNT | IT RELATES TO ME | IT RELATES TO ME COMPLETELY |
| --- | --- | --- | --- | --- | --- |
| I would like to explore strange places. | 1 | 2 | 3 | 4 | 5 |
| I like to do frightening things. | 1 | 2 | 3 | 4 | 5 |
| I like new and exciting experiences, even if I have to break the rules. | 1 | 2 | 3 | 4 | 5 |
| I prefer friends who are exciting and unpredictable. | 1 | 2 | 3 | 4 | 5 |

**Frequency of SEM use (last six months)**

How often have you used pornography during the last six months?

1 – Not once

2 – Several times

3 – Once a month

4 – 2-3 times a month

5 – Once a week

6 – Several times a week

7 – Every day or almost every day

8 – Several times a day

**Sexual experience (branching question)**

Did you have a sexual intercourse?

1 – Yes

2 – No (skip questions ##)

**Number of sexual partners (if sexually active)**

In total, with how many different people did you have a sexual intercourse since your first time?

_____ partners

**Condom use at most recent sexual intercourse (if sexually active)**

Have you and your partner used condom at most recent sexual intercourse?

1 – Yes

0 – No
